# PRS-on-Spark (PRSoS): a novel, efficient and flexible approach for generating polygenic risk scores

**DOI:** 10.1186/s12859-018-2289-9

**Published:** 2018-08-08

**Authors:** Lawrence M. Chen, Nelson Yao, Elika Garg, Yuecai Zhu, Thao T. T. Nguyen, Irina Pokhvisneva, Shantala A. Hari Dass, Eva Unternaehrer, Hélène Gaudreau, Marie Forest, Lisa M. McEwen, Julia L. MacIsaac, Michael S. Kobor, Celia M. T. Greenwood, Patricia P. Silveira, Michael J. Meaney, Kieran J. O’Donnell

**Affiliations:** 1 Douglas Hospital Research Centre, McGill University, H4H1R3, Montreal, Quebec, Canada; 2 Ludmer Centre for Neuroinformatics and Mental Health, McGill University, Montreal, QC Canada; 3 Lady Davis Institute for Medical Research, Jewish General Hospital, Montreal, Quebec, Canada; 4 Centre for Molecular Medicine and Therapeutics, University of British Columbia, Vancouver, BC Canada; 5 Department of Epidemiology, Biostatistics and Occupational Health, McGill University, Montreal, Quebec, Canada; 6 Department of Human Genetics, McGill University, Montreal, Quebec Canada; 7 Department of Oncology, McGill University, Montreal, Quebec, Canada; 8 Department of Psychiatry, McGill University, Montreal, Quebec, Canada; 9 Sackler Program for Epigenetics & Psychobiology, McGill University, Montreal, Quebec, Canada; 10 Child and Brain Development Program, Canadian Institute for Advanced Research (CIFAR), Toronto, ON Canada; 11 Singapore Institute for Clinical Sciences, Agency for Science, Technology and Research (A*STAR), Singapore, Singapore

**Keywords:** PRS-on-spark, PRSoS, Polygenic risk score, Genetic profile score, Multi-core processing, Bioinformatics, Major depressive disorder

## Abstract

**Background:**

Polygenic risk scores (PRS) describe the genomic contribution to complex phenotypes and consistently account for a larger proportion of variance in outcome than single nucleotide polymorphisms (SNPs) alone. However, there is little consensus on the optimal data input for generating PRS, and existing approaches largely preclude the use of imputed posterior probabilities and strand-ambiguous SNPs i.e., A/T or C/G polymorphisms. Our ability to predict complex traits that arise from the additive effects of a large number of SNPs would likely benefit from a more inclusive approach.

**Results:**

We developed PRS-on-Spark (PRSoS), a software implemented in Apache Spark and Python that accommodates different data inputs and strand-ambiguous SNPs to calculate PRS. We compared performance between PRSoS and an existing software (PRSice v1.25) for generating PRS for major depressive disorder using a community cohort (*N* = 264). We found PRSoS to perform faster than PRSice v1.25 when PRS were generated for a large number of SNPs (~ 17 million SNPs; *t* = 42.865, *p* = 5.43E-04). We also show that the use of imputed posterior probabilities and the inclusion of strand-ambiguous SNPs increase the proportion of variance explained by a PRS for major depressive disorder (from 4.3% to 4.8%).

**Conclusions:**

PRSoS provides the user with the ability to generate PRS using an inclusive and efficient approach that considers a larger number of SNPs than conventional approaches. We show that a PRS for major depressive disorder that includes strand-ambiguous SNPs, calculated using PRSoS, accounts for the largest proportion of variance in symptoms of depression in a community cohort, demonstrating the utility of this approach. The availability of this software will help users develop more informative PRS for a variety of complex phenotypes.

**Electronic supplementary material:**

The online version of this article (10.1186/s12859-018-2289-9) contains supplementary material, which is available to authorized users.

## Background

Polygenic risk scores (PRS) provide an index of the cumulative contribution of common variants to complex traits [1]. The approach has been applied to a large number of phenotypes, including height [2], body mass index [3], and disease risk, most notably in the prediction of psychiatric disorders [4–6]. PRS build on large existing discovery genome-wide association studies (GWAS), such as those provided by the Psychiatric Genomics Consortium (PGC) [7], which provide weights (odds ratios for binary outcomes and beta coefficients for continuous traits) that index the association between a single nucleotide polymorphism (SNP) and a phenotype of interest. Thus PRS are given by:

1 $$ PRS=\sum \limits_{i=1\dots x}^n{\beta}_i.{G}_i $$

Where β_i_ = the natural logarithm of the odds ratio (or beta coefficient) between the “i^th^” SNP and phenotype of interest and G_i_ = allele count (e.g., 0,1,2) at the “i^th^” SNP.

**Fig. 1:**
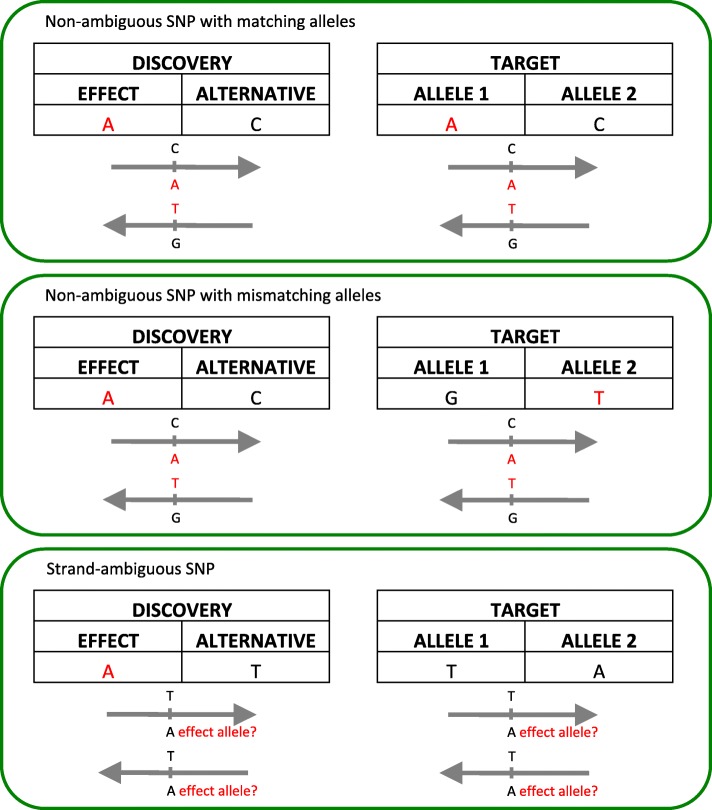
Allele matching for polygenic risk scores (PRS) between discovery and target data. The effect alleles and their reverse complements are indicated in red. Matching the effect alleles from the discovery data with the reported alleles in the target data is straightforward when SNPs are not strand-ambiguous (top and middle panel). The allele in the target data can be misassigned for strand-ambiguous SNPs (bottom)

PRS calculations are memory intensive, due to the large number of SNPs considered in each PRS. PLINK [8, 9] can be used to calculate PRS quickly. However, datasets must first undergo a series of pre-processing steps. PRSice v1.25 [10] is a software that simplifies this process through semi-automation. It is written in R and uses PLINK [8, 9] to calculate PRS. PRSice v1.25 uses observed genotypes or imputed posterior probabilities that have been converted to best guess genotypes (“hard calls”) to calculate PRS. It can also accommodate imputed posterior probabilities but relies on a dated and slower version of PLINK (version 1.07) [9]. Likewise, PRSice v1.25 discards strand-ambiguous SNPs, which are SNPs that have A/T or C/G alleles. If the strand assignment of the strand-ambiguous SNP is unknown, misassignment can occur (see Fig. 1). Many GWAS do not report the reference strand, which can lead to ambiguity on the identification of the “risk/effect allele” and the corresponding weight that should be applied. Consequently, strand-ambiguous SNPs are typically removed prior to PRS calculations (e.g., [11–15]).

As a solution we propose to use allele frequency information that many GWAS report (e.g., PGC [16, 17], GIANT [18], STARRS [19]) to identify the “effect” allele across datasets. The inclusion of the strand-ambiguous SNPs would allow researchers to retain as much information as possible from the discovery data, and likely give rise to a better understanding of the complex phenotypes.

We have developed a new software package, PRS-on-Spark (PRSoS), which accommodates observed genotypes or imputed posterior probabilities. Further, it includes a novel function that retains strand-ambiguous SNPs by using allele frequency data to identify the effect allele between discovery and target datasets. Here we test the performance of PRSoS against PRSice v1.25 using genetic data derived from a Canadian cohort and demonstrate the enhanced predictive power of PRS generated from PRSoS in the prediction of symptoms of depression.

## Implementation

PRS-on-Spark (PRSoS: https://github.com/MeaneyLab/PRSoS) is implemented in Apache Spark 2.0.0+ (Spark) and Python 2.7. Spark is an open source cluster-computing framework for big data processing that can be integrated into Python programming. As such, Spark facilitates data partitioning and parallel processing across multiple nodes and cores. For the current analyses we ran PRSoS on Linux CentOS 7, 24-core Intel Xeon server with 256GB RAM, using Spark standalone mode and a distributed file system (Apache Hadoop) with 12 cores across one worker (maximum available RAM = 48GB). PRSoS can also be implemented as a standalone version on a single cluster. PRSoS runs on the command line in Terminal on Linux or Mac, or Command Prompt in Windows. PRSoS is currently compatible with both Oxford genotype files (.gen/ .sample) and Variant Call Format (VCF) files.

Equation 2 describes our approach to PRS calculation that accommodates imputed posterior probabilities typical of imputed genetic data:

2 $$ PRS=\sum \limits_{i=1\dots x}^n\Big(2{\beta}_i.p\left({AA}_i\right)+1{\beta}_i.p\left({AB}_i\right)+0{\beta}_i.p\left({AA}_i\right) $$

Which gives:

$$ PRS=\sum \limits_{i=1\dots x}^n\left(2{\beta}_i.p\left({AA}_i\right)+{\beta}_i.p\left({AB}_i\right)\right) $$

Which gives:

$$ PRS=\sum \limits_{i=1\dots x}^n{\beta}_i.\left(2p\left({AA}_i\right)+p\left({AB}_i\right)\right) $$

where p(AA_i_*)* is the probability of a homozygous genotype for the effect allele (AA) at the i^th^ SNP, p(AB_i_) is the probability of a heterozygous genotype with one copy of the effect allele at the i^th^ SNP and p(BB_i_*)* is the probability of a homozygous genotype with zero copies of the effect allele i.e., BB genotype. This approach (Eq. 2) can also be used with observed genotypes and hard calls to calculate PRS. In the final step PRS are divided by the SNP count to obtain the weighted average across the number of SNPs called for each sample, which can vary across participants. These SNP counts for each sample are also provided in the PRSoS output.

**Fig. 2:**
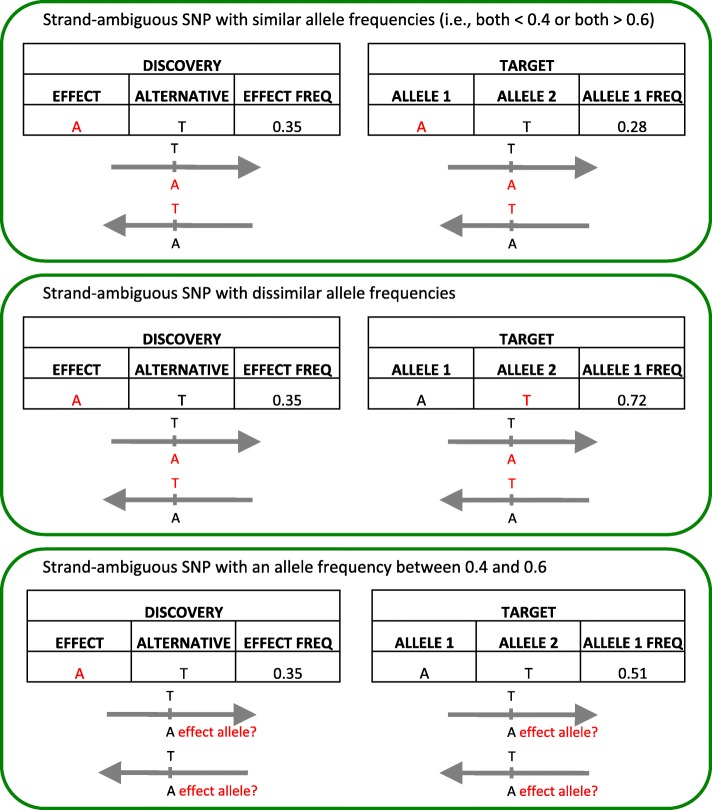
PRSoS allele matching solution for strand-ambiguous SNPs. The effect alleles and their reverse complements are indicated in red. The discovery effect allele and the target allele 1 are the same if their allele frequencies are both less than 0.4 or both more than 0.6 (top). The target allele 1 is not the effect allele if one has low allele frequency and the other has high allele frequency (middle). Strand-ambiguous SNPs with an allele frequency between 0.4 and 0.6 are excluded to increase the certainty of matching alleles

PRSoS implements an allele frequency function to match the target dataset’s allele to the effect allele reported in the discovery GWAS for ambiguous SNPs i.e., A/T or C/G allelic pairs. Note, this function does not explicitly identify strandedness (e.g., forward nor reverse strand) or perform strand alignment, rather it tests if the allele frequency of the effect allele (in the discovery dataset) matches the allele frequency for a given allele in the target dataset. The function will discard strand-ambiguous SNPs with an allele frequency between 0.4 and 0.6, to ensure alleles can be matched with a high degree of certainty. If both the allele frequencies are less than 0.4, then the first allele in the target data is scored. Likewise, if both the allele frequencies are greater than 0.6, then the first allele in the target data is scored. In contrast, if only one of the allele frequencies is less than 0.4 (while the other is greater than 0.6) then the second allele in the target data is scored (Fig. 2). This setting can be disabled (e.g., when the allele frequency in the discovery data is not provided). We note that this approach is best suited to discovery/target datasets that have a similar population structure and should not be applied to datasets with marked differences in ethnicity across cohorts.

PRSoS can also provide a SNP log documenting the SNPs included in a PRS at any given *p*-value threshold (Table 1). This SNP list can be used for subsequent gene ontology, pathway, or network analysis. The SNP log also identifies SNPs that have been excluded from the PRS, for example, when alleles in the discovery data and the target data are not identical, such as for multi-allelic SNPs.

**Table 1:** PRSoS optional data output

PRS_0.001	PRS_0.001_flag	PRS_0.5	PRS_0.5_flag	Discard
rs1115507	A1	rs1115507	A1	rs2503243
rs17692694	A2	rs11661323	A2	rs519113
rs4544201	A2	rs12296077	A1	
rs6683133	A1	rs12611811	A1	
rs7609940	A2	rs17024456	A1	
rs7620685	A1	rs17692694	A2	
		rs4544201	A2	
		rs6683133	A1	
		rs7609940	A2	
		rs7620685	A1	

Example of the SNP log included in the PRSoS output. The SNP log records the SNPs that are used in the PRS at each p-value threshold and whether the first allele column (“A1”) or the second allele column (“A2”) in the target data was scored. SNPs are recorded in the Discard column if the SNPs are discarded due to non-matching alleles between the discovery and the target data

### Sample data and polygenic risk scoring

We used genotype and phenotype data from the Maternal Adversity, Vulnerability and Neurodevelopment (MAVAN) study [20] as our target dataset. Details about the inclusion, selected measures, genotyping, quality control, and imputation are described in Additional file 1. Depressive symptoms were assessed using a well-validated, standardized questionnaire [21]. Table 2 provides a summary of the subsample used. We used the PGC major depressive disorder (MDD) GWAS as our discovery data [16]. We used the PGC MDD clumped file (pgc.mdd.clump.2012–04.txt) for all analyses. Clumping uses a greedy algorithm to selectively prune SNPs within regions of linkage disequilibrium based on the association *p*-value between each SNP and the phenotype e.g., MDD of interest [22].

**Table 2:** Maternal Adversity, Vulnerability and Neurodevelopment (MAVAN) cohort demographics. Symptoms of depression were assessed using the Center for Epidemiological Studies – Depression (CES-D) scale

Cohort Demographics	
Sample size	
Genotyping data only (used in software performance test)	N = 264
Genotyping data with symptoms of depressive score (CES-D)	*N* = 236
Mean age at time of assessment in years (SD)	34.65 (4.89)
Mean symptoms of depressive score (SD)	10.07 (8.81)
Reported ethnicity among sample with genotyping data and CES-D data	
Caucasian	*N* = 201
Others	*N* = 34
Not reported	N = 1

### Performance analysis

We compared the performance (processing times in seconds) of PRSice v1.25 and PRSoS across three types of data input: 1) imputed posterior probabilities (Imputed PP); 2) imputed genotypes converted to hard calls (Imputed HC), and 3) observed genotypes (Array Data). However, PRSice v1.25 and PRSoS are best-suited for different file formats: PLINK (.bed/.bim/.fam) format and Oxford (.gen/.sample) format, respectively. Further, .bed/.bim/.fam files are not compatible with imputed posterior probabilities. Therefore, we first compared PRSice v1.25 and PRSoS using the same format (Oxford files) for the Imputed PP. Thereafter, we compared PRSice v1.25 and PRSoS using their optimal formats for the other two data inputs (PRSice = .bed/.bim/.fam and PRSoS = .gen/.sample). We used PRSoS and PRSice v1.25 to calculate PRS at five *p*-value thresholds (P_T_ = 0.1, 0.2, 0.3, 0.4, 0.5) in a single run for each data input. Strand-ambiguous SNPs were not considered in this test. We performed this calculation three times for each software. We used a paired t-test to describe differences in total processing time. In addition, we tested if the optional SNP log available in PRSoS (see Table 1) significantly increases PRS computation time. Table 3 provides a summary of the genotype data input. All PRSoS calculations were performed using 12 physical cores in our server, with one thread of execution per core. PRSice v1.25 does not have a multi-thread option; thus it used one thread on one core. In a supplementary analysis, we illustrate the enhanced performance of PRSoS across an increasing number of cores (see Additional file 2: Fig. S1).

**Table 3:** Genotyping file information

		Genotyping file format	File size (GB)	SNP count
PRSice v1.25	Array Data	.bim/.bed/.fam	0.03	316,480
	Imputed HC	.bim/.bed/.fam	1.66	17,434,284
	Imputed PP	.gen/.fam	29.02	17,434,284
PRSoS	Array Data	.gen/.sample	0.51	316,480
	Imputed HC	.gen/.sample	28.09	17,434,284
	Imputed PP	.gen/.sample	29.02	17,434,284

The file size and SNP count provide an idea of how much data processing needs to be done by each software in our analysis. The file formats that we used in PRSice and PRSoS are different due to differences in file compatibility. All files have the same sample size (N = 264)

We also tested the performance of PRSice v1.25 and PRSoS at an increasing number of *p*-value thresholds. Specifically, we used the Imputed HC to generate PRS at 5, 10, 25, 50, 100, 125, or 200 *p*-value thresholds (P_T_ range: 0–0.5). See Additional file 1 for the executable commands used for these comparisons. Additional optional features in PRSice v1.25 (i.e., clumping and regression analysis) and PRSoS (i.e., the SNP log) were disabled in the tests to ensure that the comparisons focused solely on PRS computation.

Finally, we used PRSoS and each of the three datasets (i.e., Imputed PP, Imputed HC, Array Data) to test if the inclusion of strand-ambiguous SNPs increased the predictive value of PRS for MDD. We used linear models and compared the proportion of variance explained by PRS with and without strand-ambiguous SNPs.

## Results

**Fig. 3:**
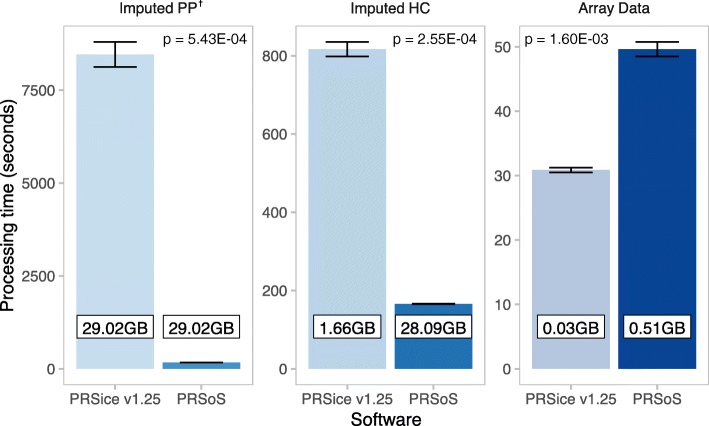
PRSice v1.25 and PRSoS performance across datasets. Bar plot shows the results of the performance test comparing running PRSice v1.25 and PRSoS across the datasets. Error bars indicate standard deviations. Numbers in boxed inserts indicate the size of the genotype data input. ^†^Note that the file sizes used for the Imputed PP are same for PRSice v1.25 and PRSoS, thus illustrating the processing speed difference with same file size input. Imputed PP = imputed posterior probabilities, Imputed HC = imputed posterior probabilities converted to “hard calls”, Array Data = observed genotypes. Significance values derived from paired t-tests

PRSoS calculated PRS (P_T_ = 0.1, 0.2, 0.3, 0.4, 0.5) using the Imputed PP in 169.6 s (SD = 0.93 s). The same calculation using PRSice v1.25 took 8461.3 s (SD = 334.6 s), which was significantly longer than PRSoS (*t* = 42.865, *p* = 5.43E-04, two-tailed; Fig. 3). Figure 3 also shows the performance of PRSice v1.25 and PRSoS using the Imputed HC and Array Data. PRSoS calculated PRS more quickly than PRSice v1.25 when using the Imputed HC (*t* = 62.627, *p* = 2.55E-04, two-tailed) but not when using the smaller Array Data (*t* = − 24.978, *p* = 1.60E-03, two-tailed), where PRSice v1.25 performed best. The addition of the SNP log output did not significantly increase processing times.

### The number of *p*-value thresholds affects PRSoS performance

**Fig. 4:**
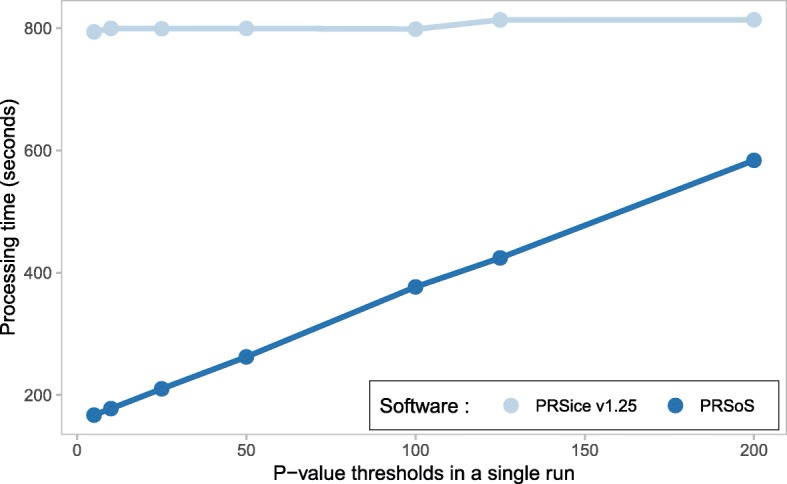
PRSice v1.25 and PRSoS performance across increasing number of *p*-value thresholds. Line plot shows the results of the performance test comparing PRSice v1.25 and PRSoS across increasing number of p-value thresholds to construct in a single run using a dataset based on imputed posterior probabilities converted to “hard calls” (Imputed HC)

PRSice v1.25 provides a “high-resolution” option, creating PRS at a large number of p-value thresholds in a single run. We tested the performance of PRSoS against PRSice v1.25 at different resolutions (up to 200 *p*-value thresholds) using the Imputed HC. PRSice v1.25 took 0.09 s (SD = 0.07 s) to calculate PRS for each threshold in addition to 795.7 s (SD = 6.6 s) for other processing operations (e.g., reading data). PRSoS processing times increased linearly with the number of thresholds (intercept = 156.8 s, slope = 2.14 s/threshold). PRSoS took 2.14 s (SD = 0.04 s) to calculate PRS for each additional threshold in addition to 156.8 s (SD = 4.1 s) for other processing operations. Although PRSoS took longer to calculate PRS for a single threshold, PRSoS calculated PRS more quickly than PRSice v1.25 in all other comparisons (Fig. 4).

### Strand-ambiguous SNPs explain additional variance in phenotype

**Fig. 5:**
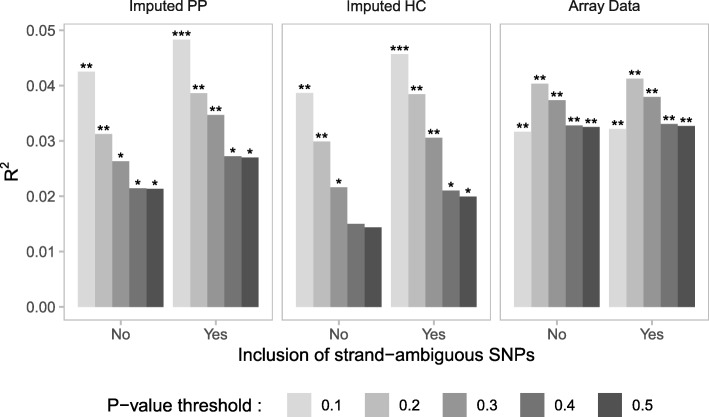
A PRS for major depressive disorder (MDD) predicts symptoms of depression. Bar plots show the proportion of variance explained by PRS for MDD in the prediction of symptoms of depression. PRS were calculated across three datasets including or excluding strand-ambiguous SNPs at a range of p-value thresholds (P_T_ = 0.1, 0.2, 0.3, 0.4, and 0.5). *p < 0.05, **p < 0.01, ***p < 0.001. Imputed PP = imputed posterior probabilities, Imputed HC = imputed posterior probabilities converted to “hard calls”, Array Data = observed genotypes

**Fig. 6:**
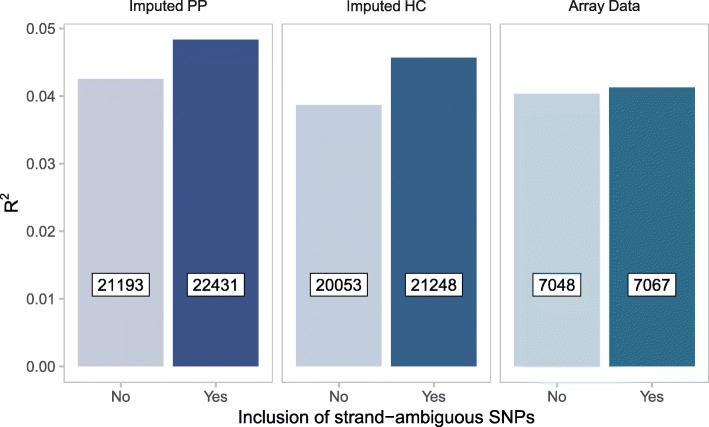
Best-fit PRS model selection. Bar plots show the proportion of variance in depressive symptoms explained by PRS for major depressive disorder (MDD) as a function of dataset with and without strand-ambiguous SNPs. Only the best-fit models are shown (P_T_: Imputed PP = 0.1, Imputed HC = 0.1, Array Data = 0.2). Numbers in boxed inserts refer to the number of SNPs included in each PRS. Imputed PP = imputed posterior probabilities, Imputed HC = imputed posterior probabilities converted to “hard calls”, Array Data = observed genotypes

We sought to determine the optimal data input (i.e., Array Data, Imputed HC, and Imputed PP datasets with and without strand-ambiguous SNPs) to derive PRS that accounted for the largest proportion of variance in symptoms of MDD. We observed a positive association between PRS for MDD and depressive symptoms across all datasets (Fig. 5) however the “best-fit” *p*-value threshold varied across different datasets. For example, the PRS at P_T_ = 0.2 accounted for the largest proportion of variance of all PRS generated from the Array Data. In contrast, the PRS at P_T_ = 0.1 performed best for both the Imputed HC and Imputed PP. PRS generated from the Imputed PP that included strand-ambiguous SNPs accounted for the largest proportion of variance in depressive symptoms (R^2^ = 0.048, F (1,234) = 11.88, *p* = 6.71E-04). In all models, the inclusion of strand-ambiguous SNPs increased the proportion of variance explained by PRS for MDD (Fig. 6).

## Discussion

PRS-on-Spark (PRSoS) is a flexible and efficient software for generating PRS. We show that PRSoS, which makes use of parallel computing, outperforms PRSice v1.25 when using imputed posterior probabilities (Imputed PP) at a number of *p*-value thresholds. We also show that PRSoS accommodates strand-ambiguous SNPs, which increase the proportion of variance explained by a PRS for MDD.

This is the first report to date comparing different strategies for calculating PRS for MDD. Our analyses demonstrate that a PRS based on imputed posterior probabilities, which includes strand-ambiguous SNPs, is the most informative predictor of symptoms of depression. Our findings also highlight the need for a more inclusive approach when generating polygenic risk predictors. This inclusive approach requires allele frequency information from the discovery GWAS. Allele frequency can vary across cohorts with different population structure, thus we recommend that this feature is used across datasets with comparable population structure [13]. We also encourage colleagues to provide allele frequency information in GWAS summary statistics rather than allele frequencies from reference datasets e.g., 1000 genomes project, which will facilitate the more accurate identification of effect alleles across datasets [23, 24].

While PRSoS outperformed PRSice v1.25 for a number of comparisons we do note that PRSice v1.25 calculated PRS more quickly when using the smallest dataset of observed genotypes (Array Data). The difference in performance between PRSice v1.25 and PRSoS when using the array data may reflect the “overhead” i.e., the time taken to parallelize the analysis of PRS when using PRSoS. Likewise, the difference in input file sizes between PRSice v1.25 (.bed file size = 0.03GB) and PRSoS (.gen file size = 0.51GB) may also contribute to the differences in performance using the Array Data. We note that PRSice v1.25 shows consistent performance across all numbers of *p*-value thresholds and is likely to outperform PRSoS when generating PRS at higher resolution (e.g., > 200 p-value thresholds). This crossing point likely varies depending on the availability of computational resources (e.g., number of cores and nodes, available memory) and the input data (e.g., file type, number of samples and SNPs). The recent beta release of PRSice-2 written in C++ improves the performance of PRSice [10] to a level somewhat comparable to PRSoS (see Additional file 3: Figure S2). Despite the enhanced performance of PRSice-2, this new software does not provide the allele frequency function available within PRSoS, which helps to identify effect alleles from stand-ambiguous SNPs across discovery and target datasets.

## Conclusions

We have developed a new software that makes use of parallel computing to accelerate PRS calculation. The increased efficiency of PRSoS and its inclusive approach to strand-ambiguous SNP together with its SNP data output will facilitate the application of PRS to better understand the polygenic basis of complex traits.

## Availability and requirements

**Project name**: PRS-on-Spark

**Project home page**: https://github.com/MeaneyLab/PRSoS

**Operating systems**: platform independent (tested on Linux CentOS 7 server and Ubuntu 16, MacOS Sierra, and Microsoft Windows 10 standalone computers)

**Programming language**: Python

**Other requirements**: Python 2.7, Spark 2.0.0 or higher, Scala 2 or higher, Java 7 or higher, Hadoop 2.6 or higher, Python modules (matplotlib, statsmodels, pandas, numpy)

**License**: GNU GPL v3, Apache License 2.0

**Any restrictions to use by non-academics**: None

## Additional files

Additional file 1: Supplementary Methods and Data Analysis. Additional information on the MAVAN cohort. Genetic data quality control and supplementary data analyses are provided. (Additional file 4: Figure S3, Additional file 5: Figure S4). (PDF 142 kb)

Additional file 2: **Figure S1.** PRSice v1.25 and PRSoS performance across the number of cores used to generate PRS and five thresholds using the Imputed Hard Call dataset. PRSice v1.25 could only run on 1 core. PRSoS performance was tested with 1, 4, 12, 20, and 24 cores on a Linux CentOS 7, 24-core Intel Xeon server. Error bars indicate standard deviations. (PDF 4 kb)

Additional file 3: **Figure S2.** PRSice v1.25, PRSice-2, and PRSoS performance across datasets. Bar plot shows the results of the performance test comparing running PRSice v1.25, PRSice-2, and PRSoS across the datasets. Processing time (y-axis) uses a log base 10 scale. Error bars indicate standard deviations. Numbers in boxed inserts indicate the size of the genotype data input. ^†^Note that the file sizes used for the Imputed PP are same for PRSice v1.25 and PRSoS, thus illustrating the processing speed difference with same file size input. Genotype input formats are different across all three software for the other performance tests. Imputed PP = imputed posterior probabilities, Imputed HC = imputed posterior probabilities converted to “hard calls”, Array Data = observed genotypes. (PDF 34 kb)

Additional file 4: **Figure S3.** Software performance of generating PRS at five *p*-value thresholds in a single run with different sample sizes. The left panel shows the results using the Imputed Hard Call dataset (*N* = 264). The right panel shows the results using simulated data based on the Imputed Hard Call dataset with five times the sample size (*N* = 1320). Error bars indicate standard deviations. (PDF 4 kb)

Additional file 5: **Figure S4.** Software performance between datasets across number of PRS p-value thresholds to generate in a single run. Imputed HC = imputed posterior probabilities converted to “hard calls”, Array Data = observed genotypes. (PDF 5 kb)

## Abbreviations

Array Data: Observed genotype dataset
GWAS: Genome-wide association study
Imputed HC: Imputed hard call dataset
Imputed PP: Imputed posterior probability dataset
MAVAN: Maternal Adversity, Vulnerability and Neurodevelopment
MDD: Major depressive disorder
PGC: Psychiatric Genomics Consortium
PRS: Polygenic risk scores
PRSoS: PRS-on-Spark
P_T_: *P*-value threshold
SD: Standard deviation
SNP: Single nucleotide polymorphism
Spark: Apache Spark
