# Supplementary material for: PRS-on-Spark (PRSoS): a novel, efficient and flexible approach for generating polygenic risk scores
Source: BMC Bioinformatics. 2018 Aug 8;19:295. doi: 10.1186/s12859-018-2289-9 (PMC6083617; doi:10.1186/s12859-018-2289-9)
Supplement: Supplementary file 1 — Supplementary Methods and Data Analysis. Additional information on the MAVAN cohort. Genetic data quality control and supplementary data analyses are provided. (Additional file 4: Figure S3, Additional file 5: Figure S4). (PDF 142 kb) [file 12859_2018_2289_MOESM1_ESM.pdf]

Supplementary Methods

Cohort:

The sample comprised of mothers participating in the Maternal Adversity, Vulnerability and Neurodevelopment (MAVAN) Project. MAVAN is an ongoing, longitudinal, birth cohort study that has recruited over 500 mother-child dyads in Hamilton (Ontario) and Montreal (Quebec), Canada, since 2003 [1]. The project focuses on the development of individual differences in vulnerability for mental illness through the interplay of biological mechanisms and maternal adversity in early life. The families participate in surveys, interviews, social experiments, and behavioral and cognitive experiments at 0.5, 1, 1.5, 2, 3, 4, 5, 6, 8, and 10 years postpartum. Fully informed written consent was obtained from the mothers and ethics approval was obtained from the Comité d'éthique de la recherche at the Douglas Hospital Research Centre (Montreal, Canada).

The mothers reported their depressive symptoms at 4 years postpartum using the Center for Epidemiological Studies Depression Scale (CES-D) [2]. The questionnaire consists of 20 questions about their feelings and behaviors during the past week. Each question is scored between 0 and 3 with higher scores reflecting a greater burden of depressive symptoms.

A subsample of the cohort with CES-D scores and genotype data (n = 236) were used for the regression analysis. Table 1 describes the subsample used.

Genotyping, quality control, and imputation:

Participants provided buccal epithelial cells which were used to extract DNA for genomic analyses. We used the PsychChip and PsychArray (Illumina) to describe genetic variation in women (N = 264; mean age: 35 years) from the MAVAN study [1]. We used good quality (call rate >95%) genetic markers shared across the PsychChip and PsychArray platforms for our analyses (n=551,589 markers). We removed genetic variants that deviated from Hardy-Weinberg equilibrium at p<1E-13, and non-autosomal variants (e.g., indels, sex chromosome markers), resulting in 316,480 autosomal SNPs. We also checked for samples' heterozygosity rate, biological sex estimate, and cryptic relatedness (identity-by-descent) between samples. We further removed SNPs with minor allele frequencies <0.05 (resulting in 240,566 SNPs) to submit to Sanger Imputation Service to generate our imputed genotype datasets using the Haplotype Reference Consortium for reference genotypes [3]. Specifically, each chromosome was phased against the reference panel using EAGLE2 (v2.0.5) [4]. Imputed posterior probabilities then were calculated from the phased data using Positional Burrows-Wheeler Transform methods with the PBWT software [5]. We removed SNPs with imputation accuracy (INFO score) <0.30, resulting in 27,232,417 autosomal SNPs in our imputed posterior probabilities dataset to generate PRS for the regression analysis. For the performance tests, a third dataset was generated from the imputed data by removing strand-ambiguous SNPs and SNPs with an INFO score <0.80, resulted in 17,434,284 autosomal SNPs (Imputed PP). A posterior probability threshold of 0.90 was used through PLINK 1.9 [6] to convert the posterior probabilities to hard calls to generate the imputed hard call dataset (Imputed HC).

Discovery data:

The PRS calculation requires data from two independent cohorts. One cohort is the study cohort of interest (target data); we describe the genetic risk in this cohort (e.g., the MAVAN cohort presented in our study). The other cohort (discovery data) provides information about the risk associated with each SNP (i.e., the effect size and the p-value of the trait association).

We used the Psychiatric Genomics Consortium Major Depressive Disorder dataset (N = 18,759) as the discovery cohort for all our PRS calculations [7]. We used their “clumped” results, which is a subset of the full results created after pruning SNPs that are in high linkage disequilibrium ($r^2 = 0.25$ within a window of 500kb) and selectively retaining the most strongly associated SNPs in the linkage disequilibrium regions. SNPs with minor allele frequency ≤2% and INFO score <0.9 were also removed.

Examples of the executed commands for performance analysis:

All commands were executed in Terminal on Linux CentOS 7.

Timing PRSoS generating PRS at five p-value thresholds (P_T = 0.1, 0.2, 0.3, 0.5):

```
/usr/bin/time –v `# <-- this functions to time process completion` \
spark-submit PRS_run.py \
MAVAN_ArrayData.gen \
GWAS_MDD_beta_clump_noambi.txt \
PRSoS_0.1_arr \
--sample MAVAN_ArrayData.samples \
--sample_skip 2 \
--sample_delim " " \
--filetype GEN \
--gwas_delim "\t" \
--threshold_seq 0.1 0.5 0.1 \
--no_a1f \
&>> PRSoS_0.1_arr.timelog `# <-- this writes standard output and
saves it to file to record processing time`
```

Timing PRSice v1.25 generating PRS at five p-value thresholds (P_T = 0.1, 0.2, 0.3, 0.5):

```
/usr/bin/time -v \
R -q --file=PRSice_v1.25.R \
--args plink /share/bin/plink \
base GWAS_MDD_beta_clump_noambi.txt \
target MAVAN_ArrayData \
slower 0 supper 0.5 sinc 0.1 \
clump.snps F \
```

```
report.best.score.only F \
allow.no.sex T \
covary F \
pheno.file phenoNA.pheno \
no.regression T \
&>> PRSice_0.1_arr.timelog
```

Supplementary Analysis

PRSice v1.25 and PRSoS performance across number of cores used:

We tested how the number of cores used affects the processing time a PRSoS run. We ran the PRSoS software using the Imputed HC with 1, 4, 12, 20, and 24 cores, three times each. A one-way ANOVA was conducted to compare the effect of the number of cores used on processing time. There was a significant effect of the number of cores used on processing time ($F(4,10) = 119{,}253$, $p < 0.0001$). Post hoc comparisons using the Tukey's HSD method indicated that using 1 core (M = 1305.0sec, SD = 5.0sec) is significantly slower than using 4 cores (M = 387.8sec, SD = 1.6sec), using 4 cores is significantly slower than using 12 cores (M = 154.7sec, SD = 0.3sec), and using 12 cores is significantly slower than using 20 cores (M = 126.1sec, SD = 0.7sec) (Supplementary Figure 1).

As PRSice v1.25 can only run on a single core, we also compared the performance of PRSice v1.25 and PRSoS on a single core using a paired t-test. PRSice v1.25 calculated PRS significantly more quickly than PRSoS when using one core only ($t = 61.304$, $p = 2.66E-04$, two-tailed). However, PRSoS calculated PRS significantly more quickly than PRSice v1.25 when PRSoS used multiple cores (all $p < 0.001$).

PRSice-2 performance comparison:

PRSice version 2 (PRSice-2) is a recent release of PRSice [8]. The software uses binary PLINK (.bed) or binary Oxford (.bgen) input files. These files are much smaller than their plain text counterparts (i.e., .ped for PLINK and .gen for Oxford format) [9]. We compared the performance of PRSice-2 with PRSice v1.25 and PRSoS for

calculating PRS at five p-value thresholds, using the Imputed PP, Imputed HC, and Array Data. We provided .bgen input files for PRSice-2. PRSice-2 ran using one thread on one core. It outperformed the other software in all three datasets (Imputed PP: M = 94.2sec, SD = 0.2sec; Imputed HC: M = 93.2sec, SD = 1.8sec; Array Data: M = 2.6sec, SD = 0.01sec) (Supplementary Figure 2). Paired t-tests indicated that PRSice-2 completes a run with less time than PRSoS in all three datasets (Imputed PP: t = 130.147, p = 5.90E-05; Imputed HC: t = 56.348, p = 3.14E-04; Array Data: t = 73.202, p = 1.87E-04; all two-tailed).

The number of SNPs within the target dataset influences PRSoS performance:

In our examples, the Array Data (316,480 SNPs, 264 samples) took 49.6sec (SD = 1.1sec) to run using PRSoS. The Imputed HC (17,434,284 SNPs, 264 samples) took 165.9sec (SD = 0.4sec) to run under the same environment. A paired t-test indicated that the difference is significant (t = 148.7, p = 4.52E-05, two-tailed). Figure 3 illustrates the difference.

Influence of sample size on PRSoS performance:

We simulated sample data by replicating the Imputed HC's sample data fivefold (17,434,284 SNPs, simulated n=1320 samples) and calculated PRS using PRSoS three times. Specifically, we ran PRSoS three times to calculate PRS at five p-value thresholds (P_T range: 0-0.5, interval: 0.1). A paired t-test indicated that using the larger sample set (M = 558.4sec, SD = 31.2sec) takes significantly longer time to run than using the Imputed HC (t = 21.835, p = 2.09E-03, two-tailed). We also ran PRSice v1.25 with the

larger simulated dataset under similar conditions (using the binary PLINK file input instead) to provide a reference point. A paired t-test indicated no significant difference in PRSice v1.25 performance between using the Imputed HC (M = 816.7sec, SD = 18.4sec) and the larger simulated dataset (M = 830.8sec, SD = 3.7sec) (t = 1.243, p = 0.34, two-tailed) (Supplementary Figure 3).

Number of p-value thresholds and SNPs influence PRSice v1.25 and PRSoS performance:

We tested the performance of PRSice v1.25 and PRSoS at an increasing number of p-value thresholds in a single run using the Imputed HC (17,434,284 SNPs, 264 samples) and Array Data (316,480 SNPs, 264 samples). Each software generated PRS at 5, 10, 25, 50, 100, 125, or 200 p-value thresholds (P_T range: 0-0.5). The processing time for the Array Data is shorter than for the Imputed HC for both software at each p-value threshold (Supplementary Figure 4). PRSoS showed a linear increase in processing time as the number of thresholds to process increased with both the Imputed HC (intercept = 156.8sec, slope = 2.14sec/threshold) and the Array Data (intercept = 45.0sec, slope = 1.38sec/threshold). PRSice v1.25 showed a mean of 802.6sec (SD = 7.8sec) for the Imputed HC and a mean of 35.2sec (SD = 1.1sec) for the Array Data for each p-value threshold set in a single run (Supplementary Figure 4).

Supplementary References

1. O'Donnell KA, Gaudreau H, Colalillo S, Steiner M, Atkinson L, Moss E, et al. The Maternal Adversity, Vulnerability and Neurodevelopment project: theory and methodology. Can J Psychiatry. 2014;59:497-508.

2. Radloff LS. The CES-D scale: a self-reported depression scale for research in the general population. Appl Psychol Meas. 1977;1:385-401.

3. McCarthy S, Das S, Kretzschmar W, Delaneau O, Wood AR, Teumer A, et al. A reference panel of 64,976 haplotypes for genotype imputation. Nat Genet. 2016;48:1279-83.

4. Loh P-R, Danecek P, Palamara PF, Fuchsberger C, A Reshef Y, K Finucane H, et al. Reference-based phasing using the Haplotype Reference Consortium panel. Nat Genet. 2016;48:1443-8.

5. Durbin R. Efficient haplotype matching and storage using the positional Burrows-Wheeler transform (PBWT). Bioinformatics. 2014;30:1266-72.

6. Chang CC, Chow CC, Tellier LCAM, Vattikuti S, Purcell SM, Lee JJ. Second-generation PLINK: rising to the challenge of larger and richer datasets. GigaScience. 2015;4:1-16.

7. Major Depressive Disorder Working Group of the Psychiatric GWAS Consortium. A mega-analysis of genome-wide association studies for major depressive disorder. Mol Psychiatry. 2013;18:497-511.

8. Euesden J, Lewis CM, O'Reilly PF. PRSice: Polygenic Risk Score software. Bioinformatics. 2014;31:1466-8.

9. Band G, Marchini J. BGEN: a binary file format for imputed genotype and haplotype data. bioRxiv. 2018; doi:10.1101/308296.

Supplementary Figure Legends:

Supplementary Figure 1. PRSice v1.25 and PRSoS performance across the number of cores used to generate PRS and five thresholds using the Imputed Hard Call dataset. PRSice v1.25 could only run on 1 core. PRSoS performance was tested with 1, 4, 12, 20, and 24 cores on a Linux CentOS 7, 24-core Intel Xeon server. Error bars indicate standard deviations.

Supplementary Figure 2. PRSice v1.25, PRSice-2, and PRSoS performance across datasets. Bar plot shows the results of the performance test comparing running PRSice v1.25, PRSice-2, and PRSoS across the datasets. Processing time (y-axis) uses a log base 10 scale. Error bars indicate standard deviations. Numbers in boxed inserts indicate the size of the genotype data input. [†]Note that the file sizes used for the Imputed PP are same for PRSice v1.25 and PRSoS, thus illustrating the processing speed difference with same file size input. Genotype input formats are different across all three software for the other performance tests. Imputed PP = imputed posterior probabilities, Imputed HC = imputed posterior probabilities converted to “hard calls”, Array Data = observed genotypes.

Supplementary Figure 3. Software performance of generating PRS at five p-value thresholds in a single run with different sample sizes. The left panel shows the results using the Imputed Hard Call dataset (N = 264). The right panel shows the results using

simulated data based on the Imputed Hard Call dataset with five times the sample size (N = 1320). Error bars indicate standard deviations.

Supplementary Figure 4. Software performance between datasets across number of PRS p-value thresholds to generate in a single run. Imputed HC = imputed posterior probabilities converted to “hard calls”, Array Data = observed genotypes.
