# Supplementary figures and images for: PRS-on-Spark (PRSoS): a novel, efficient and flexible approach for generating polygenic risk scores
Source: BMC Bioinformatics. 2018 Aug 8;19:295. doi: 10.1186/s12859-018-2289-9 (PMC6083617; doi:10.1186/s12859-018-2289-9)

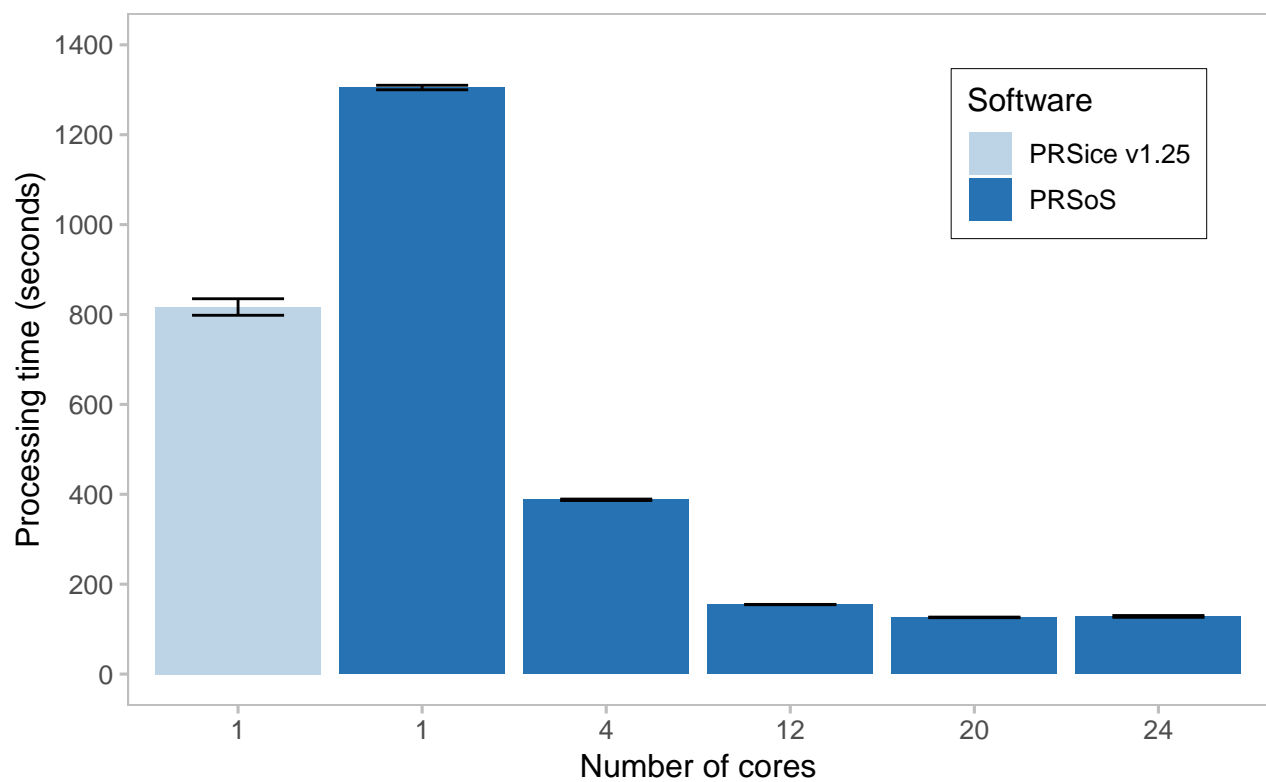

Supplement: Supplementary file 2 — Figure S1. PRSice v1.25 and PRSoS performance across the number of cores used to generate PRS and five thresholds using the Imputed Hard Call dataset. PRSice v1.25 could only run on 1 core. PRSoS performance was tested with 1, 4, 12, 20, and 24 cores on a Linux CentOS 7, 24-core Intel Xeon server. Error bars indicate standard deviations. (PDF 4 kb) [file 12859_2018_2289_MOESM2_ESM.pdf]

Processing time (seconds)

Imputed PP<sup>†</sup>

Imputed HC

Array Data

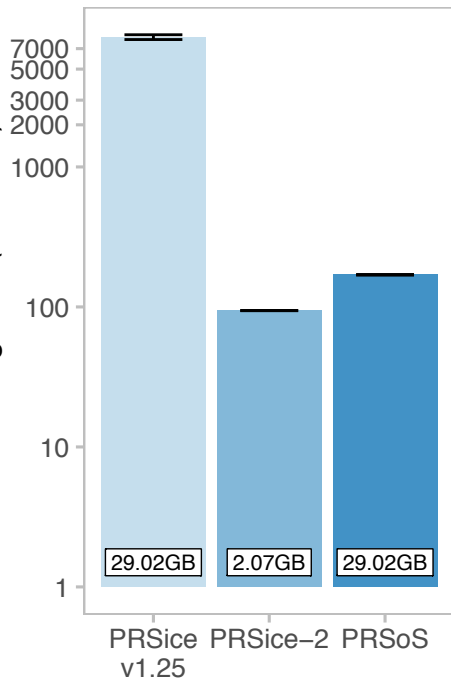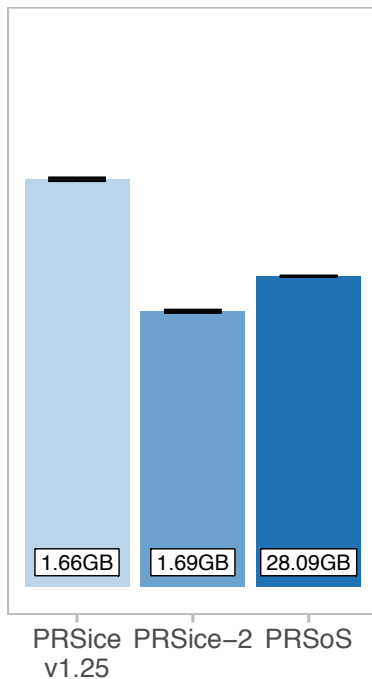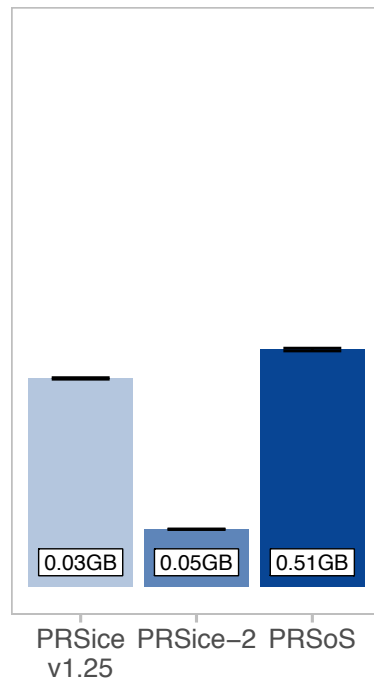

Software

Supplement: Supplementary file 3 — Figure S2. PRSice v1.25, PRSice-2, and PRSoS performance across datasets. Bar plot shows the results of the performance test comparing running PRSice v1.25, PRSice-2, and PRSoS across the datasets. Processing time (y-axis) uses a log base 10 scale. Error bars indicate standard deviations. Numbers in boxed inserts indicate the size of the genotype data input. †Note that the file sizes used for the Imputed PP are same for PRSice v1.25 and PRSoS, thus illustrating the processing speed difference with same file size input. Genotype input formats are different across all three software for the other performance tests. Imputed PP = imputed posterior probabilities, Imputed HC = imputed posterior probabilities converted to “hard calls”, Array Data = observed genotypes. (PDF 34 kb) [file 12859_2018_2289_MOESM3_ESM.pdf]

N = 264

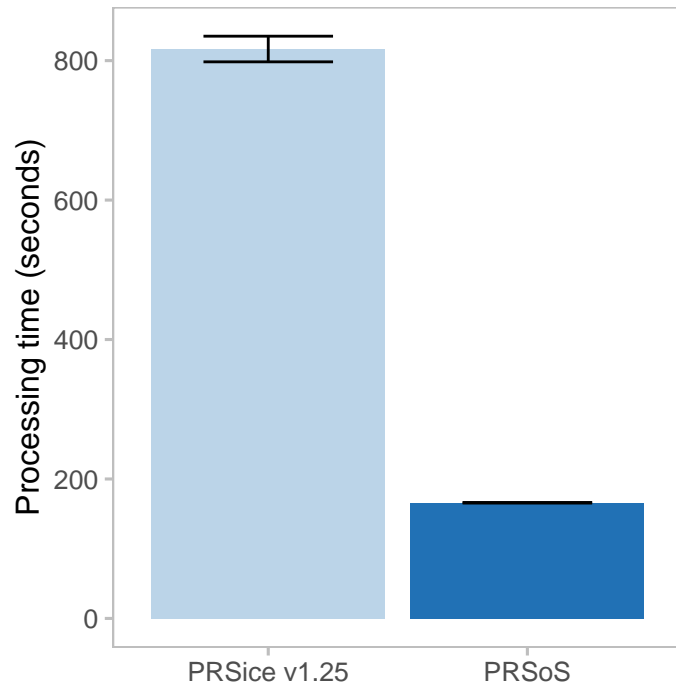

N = 1320

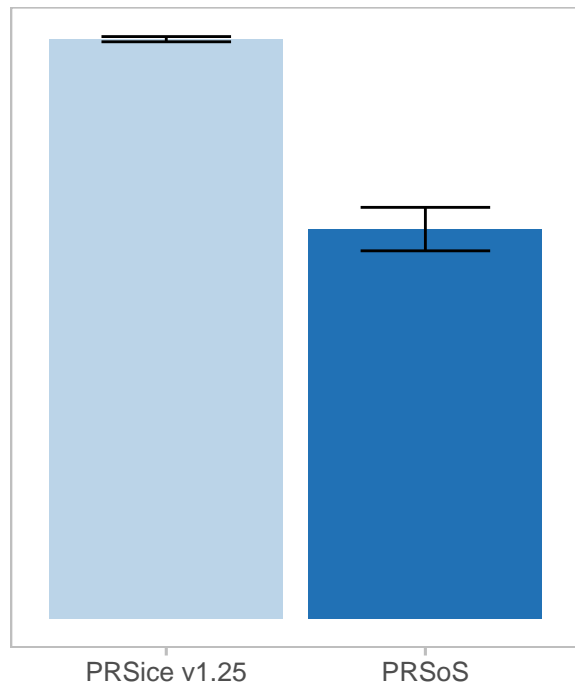

Software

Supplement: Supplementary file 4 — Figure S3. Software performance of generating PRS at five p-value thresholds in a single run with different sample sizes. The left panel shows the results using the Imputed Hard Call dataset (N = 264). The right panel shows the results using simulated data based on the Imputed Hard Call dataset with five times the sample size (N = 1320). Error bars indicate standard deviations. (PDF 4 kb) [file 12859_2018_2289_MOESM4_ESM.pdf]

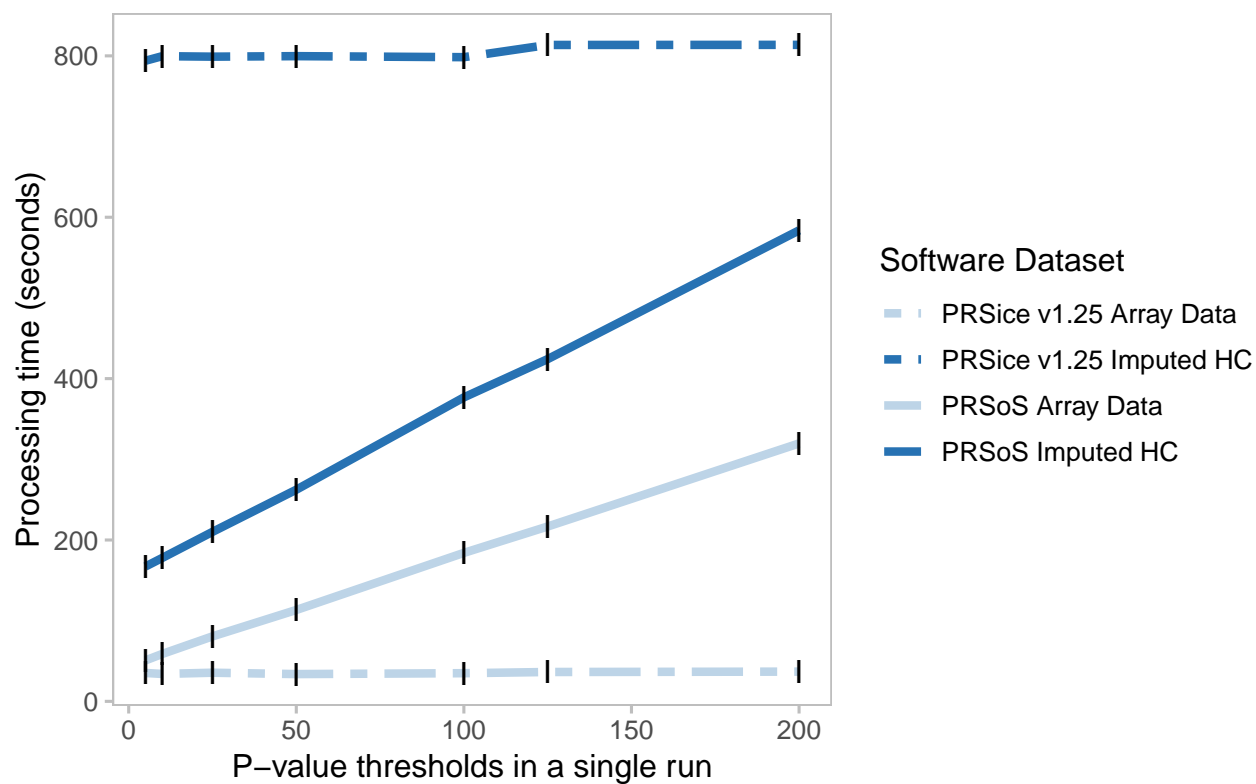

Supplement: Supplementary file 5 — Figure S4. Software performance between datasets across number of PRS p-value thresholds to generate in a single run. Imputed HC = imputed posterior probabilities converted to “hard calls”, Array Data = observed genotypes. (PDF 5 kb) [file 12859_2018_2289_MOESM5_ESM.pdf]
